# Is it a hemangioma? A combined case report of soft tissue sarcomas mimicking infantile hemangioma

**DOI:** 10.1093/jscr/rjaf748

**Published:** 2025-09-24

**Authors:** Shaden Almutairi, Wed Al Wabel, Saud Aljadaan, Norah Alsabty, Afnan Alshehri, Faten Alrobian

**Affiliations:** College of Medicine at King Saud bin Abdulaziz University for Health Sciences, Shiekh Jaber Al-Sabah Road, Khasem Ala’an, 14611 Riyadh, Saudi Arabia; College of Medicine at King Saud bin Abdulaziz University for Health Sciences, Shiekh Jaber Al-Sabah Road, Khasem Ala’an, 14611 Riyadh, Saudi Arabia; Pediatric Surgery Department, King Abdullah Specialized Children’s Hospital, Ar Rimayah, 14611 Riyadh, Saudi Arabia; Pediatric Surgery Department, King Abdullah Specialized Children’s Hospital, Ar Rimayah, 14611 Riyadh, Saudi Arabia; Pediatric Radiology Department, King Abdullah Specialized Children’s Hospital, Ar Rimayah, 14611 Riyadh, Saudi Arabia; Pediatric Surgery Department, King Abdullah Specialized Children’s Hospital, Ar Rimayah, 14611 Riyadh, Saudi Arabia

**Keywords:** hemangioma, vascular malformation, soft tissue sarcoma, neonatal tumors, pediatric surgery

## Abstract

Infantile hemangiomas (IHs) are the most common infantile tumors. They are benign and often responsive to propranolol. IHs are difficult to distinguish from malignant soft tissue tumors like sarcomas. Sarcomas are rare mesenchymal tumors that mimic hemangiomas, delaying treatment and affecting outcomes. We report two instances in which female infants with sarcomas were initially misdiagnosed as hemangiomas. In the first case, propanolol was administered to a 1-month-old presenting with a left inguinal mass. Imaging revealed a hemangioma. Tumor growth necessitated a biopsy, revealing a sarcoma. In the second case, a 2-month-old presented with a rapid growing and ulcerating left-hand swelling and was misdiagnosed as a hemangioma. Imaging and biopsy revealed metastatic sarcoma. Both patients developed brain metastases and passed away. These cases highlight that IHs need to be re-evaluated when rapid growth or ulceration is encountered. Early detection is essential for optimal outcomes.

## Introduction

Historically known as “birthmarks,” infantile hemangiomas (IHs) are the most common tumors of infancy. Approximately 5%–10% of 1-year-olds have infantile hemangiomas involving soft tissues [1]. As a result of its favorable safety profile and reported success rate > 90%, propranolol, a nonselective beta-adrenergic antagonist, became recognized as the first-line treatment for infantile hemangiomas [1]. Congenital hemangioma is a different clinical entity and differs from infantile hemangioma in the growth pattern, as the former does not proliferate after birth [2].

Most IHs are clinically diagnosed and do not need imaging for diagnosis. Radiological imaging is indicated to evaluate the extent of lesion and presurgical/interventional planning. It can be indicated in atypical clinical presentations, such as atypical morphology (ulceration, bleeding, etc.), unresponsiveness to treatment, or rapid growth. Ultrasound (US) and magnetic resonant imaging (MRI) are the two modalities of choice [3].

On gray scale US, IHs appear as heterogeneous but predominantly hyperechoic nodules with fairly well-delineated borders and presence of marked vascularity with high- and low-resistance arterial and venous waveforms on color Doppler US imaging. On MRI, IHs are seen as circumscribed nodules or plaques that are hyperintense (although less than fluid) on *T*_2_-weighted MR images and heterogeneous with fatty elements on *T*_1_-weighted MR images. Flow voids and rapid, vivid contrast enhancement are typical. Calcifications are typically not seen with IHs [4].

Malignant soft tissue tumors of children tend to be difficult to detect because of the rarity and diversity of clinical presentation, and thus misdiagnosed [5]. Management of each of these diseases differs widely, and outcomes of treatment and survival are affected by the timing of the diagnosis [6].

Here, we report two cases of hemangiomas in newborns who were treated with propranolol initially and then, due to the behavior of the lesions, were biopsied and diagnosed as sarcomas. These cases showed progressive lesion growth, unusual imaging results, and, at the end, a fatal outcome.

## Case presentation

### Case 1

**Figure 1:**
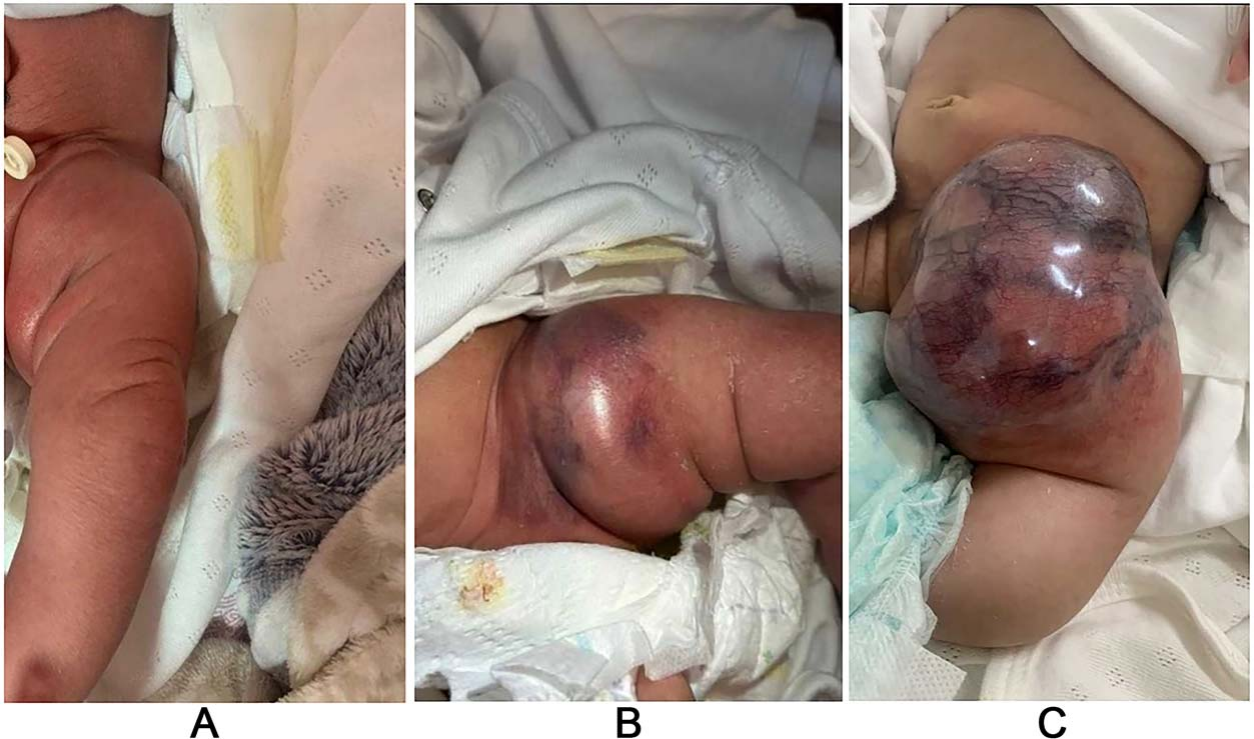
Case #1 lesion progression: (A) at birth, (B) 3 weeks prior to presentation, (C) upon presentation.

A 1-month-old female was being evaluated in an outside hospital for a lesion, which was diagnosed as a hemangioma. The patient was started on oral propranolol for treatment; however, treatment was stopped after 1 week as the mother noticed a significant increase in the size of the lesion and sought another opinion at our center. On examination, a firm, well-defined mass was seen on the left inguinal region (Fig. 1).

**Figure 2:**
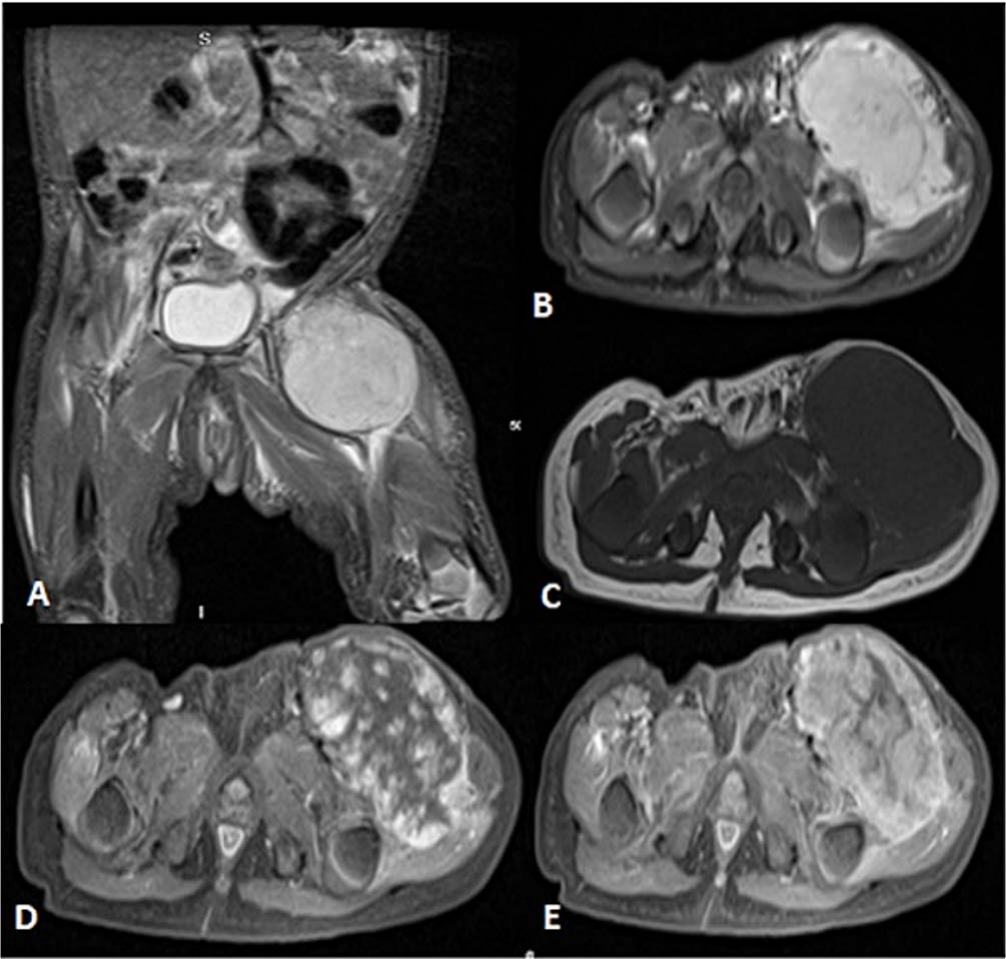
Uploaded MRIs of the left inguinal mass. (A and B) Coronal and axial short tau inversion recovery (STIR) images show a well-defined large heterogeneously high signal intensity with an area of signal void arising from the left upper thigh quadriceps muscles region. (C) Axial T1WI shows that mass appear isointense in signal intensity in correlation to the muscle signal with no intralesional hemorrhagic component. (D and E) Axial *T*_1_ fat-saturated images’ early and delayed phases show peripheral discontinuous nodular enhancement with subsequent centripetal enhancement on the delayed phase. The aforementioned MR findings are typical for hemangiomas.

**Figure 3:**
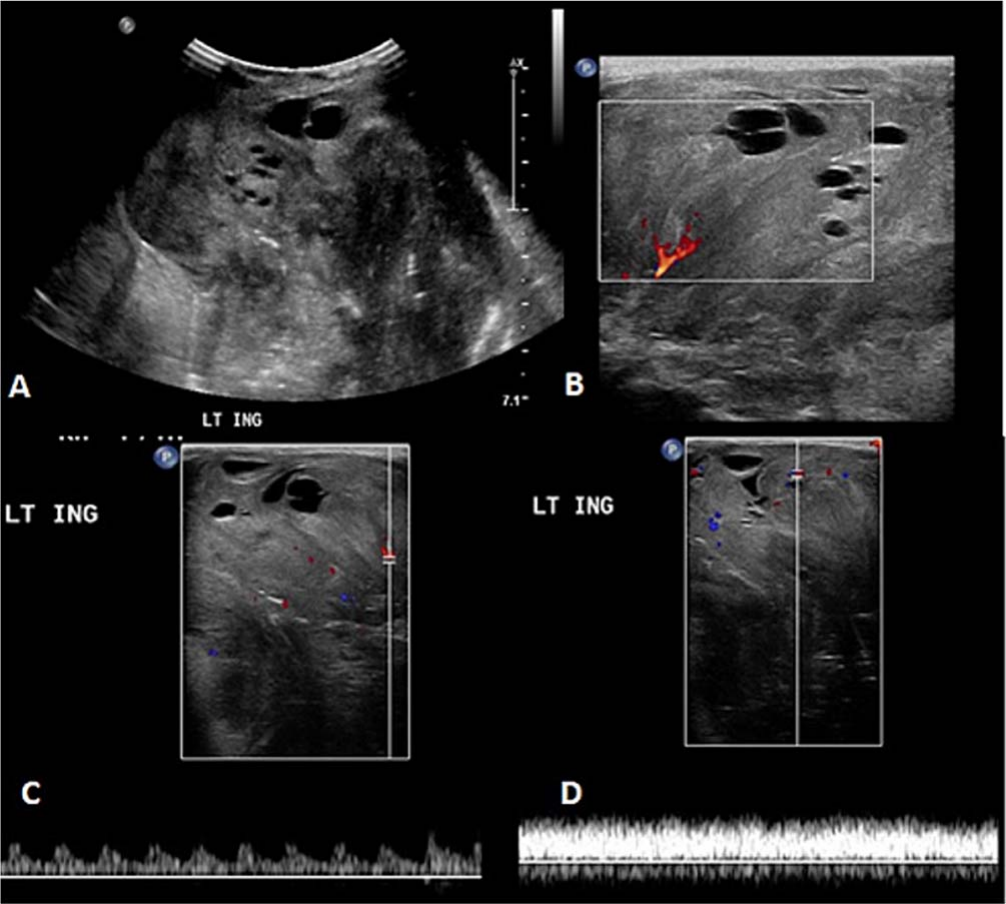
The follow-up ultrasound 3 weeks after MRI. (A) The gray scale image demonstrates a large heterogeneous predominantly isoechoic mass seen at the left inguinal region with multiple scattered areas of cystic changes and difficult-to-assess extent due to its size. (B–D) Color Doppler and spectral wave images show vascularity within the mass with detectable arterial and venous waveform.

Before the presentation, the patient had an MRI done 3 weeks prior which showed a left thigh quadriceps muscle region mass measuring 3.9 × 4 × 4.7 cm. The mass showed peripheral discontinuous nodular enhancement in the early phase with centripetal enhancement in the delayed phase on postcontrast images, matching the description of a hemangioma (Fig. 2). Upon presentation, a left inguinal US was done, which showed a mass measuring 9.1 × 8.3 × 8.7 cm. The mass appeared heterogeneously isoechoic with areas of cystic changes and showed atypical manifestation on color Doppler. The US was done 3 weeks after the MRI (Fig. 3).

A biopsy was done by interventional radiology as it is a less invasive method. It showed a round blue cell and extensive necrosis; thus, an incisional biopsy was obtained. The pathology result was a round cell sarcoma NOS (not otherwise specific). The lesion was positive for NF1 using FoundationOne Heme, a test that utilizes DNA sequencing to interrogate 406 genes and introns of 31 genes involved in rearrangements, as well as the sequencing of 265 RNA genes. Whole exome sequencing result was positive for the Neurofibromatosis type 1 gene.

**Figure 4:**
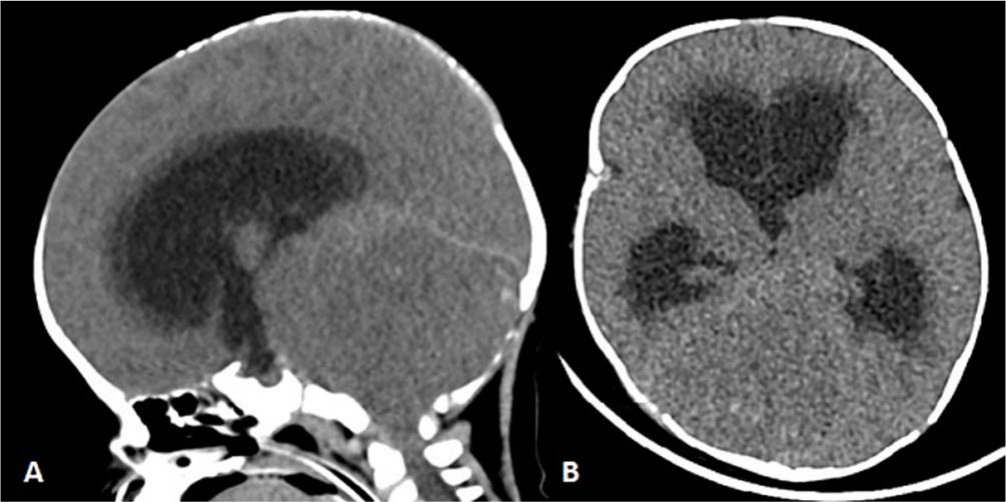
Patient underwent brain CT after presenting to the ER due to seizure. (A and B) Sagittal and axial nonenhanced brain CT shows an isodense mass in the posterior cranial fossa with scattered areas of hyperdense foci denoting a hemorrhagic component that is completely effacing the fourth ventricle, causing supratentorial ventricular dilatation and periventricular hypodensity denoting cerebrospinal fluid (CSF) permeation indicating an active obstructive hydrocephalus. Also noted bilateral tonsillar herniation.

To achieve local control, surgical management using hemipelvectomy was suggested based on the lesion’s size and progression. However, this option was refused by the family. The patient was then started on chemotherapy. Whole body staging MRI did not show any metastatic lesions. After 3 months of treatment, the patient started having generalized seizure episodes. Brain neuroimaging with computed tomography (CT) scan showed an isodense mass in the posterior fossa, which was causing tonsillar herniation and obstructive active supratentorial hydrocephalus (Fig. 4). Goals of care were determined and the patient was transitioned to comfort care, after which she passed away.

### Case 2

**Figure 5:**
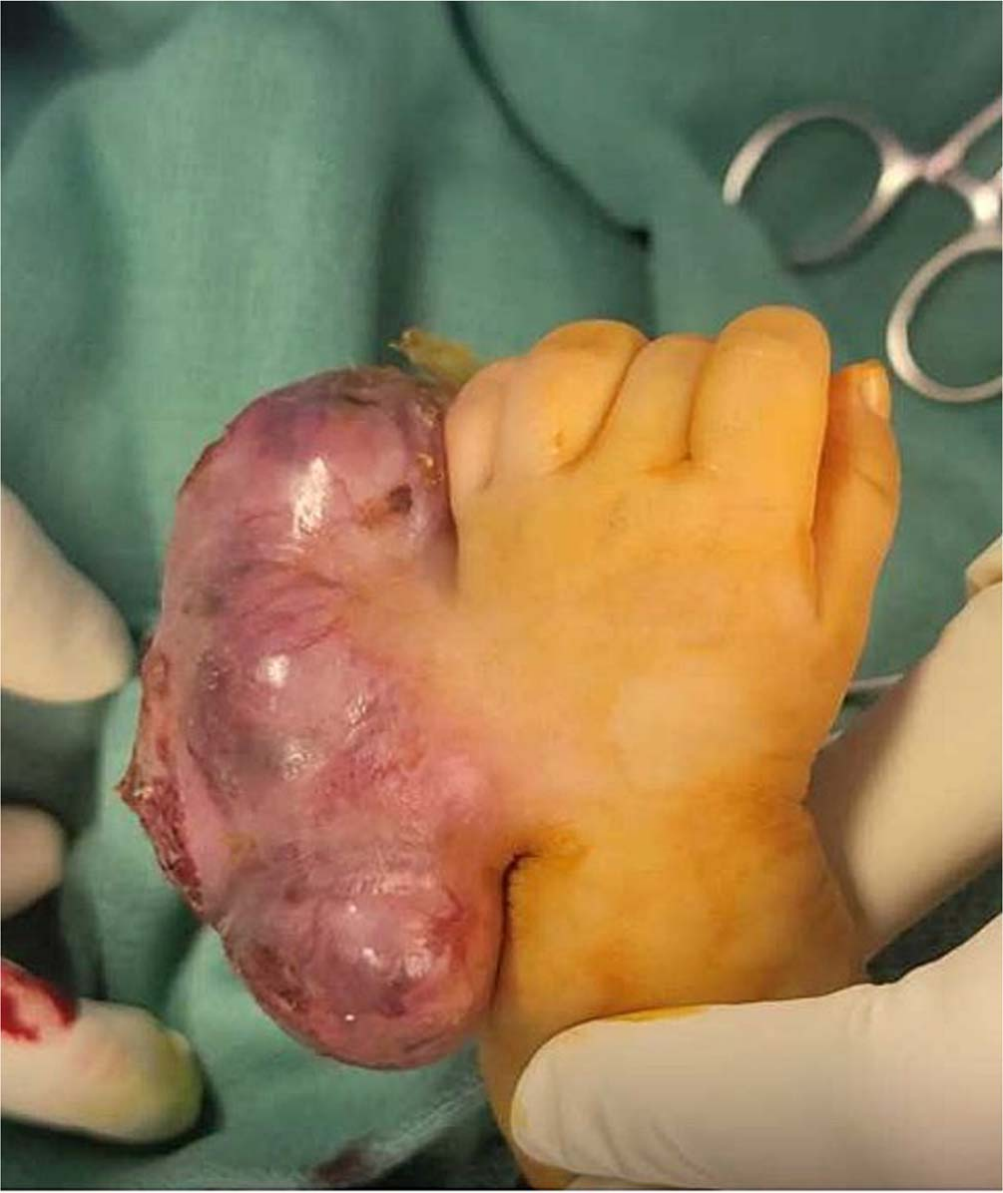
Lesion size 4 × 2 cm.

**Figure 6:**
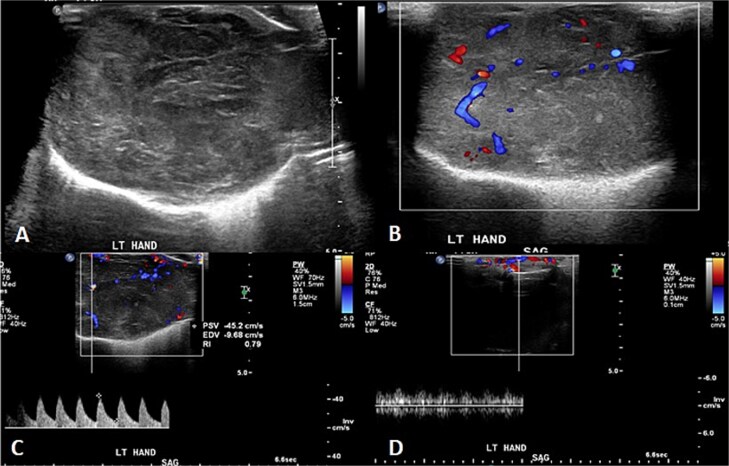
Initial imaging utilizing gray scale and color Doppler US. (A) A gray scale US image showing a large heterogeneous lesion predominantly hyperechogenic in echotexture with a partially defined margin and (B–D) color Doppler US and spectral waveform showing internal vascularity within the lesion with the presence of arterial and venous waveform. The aforementioned findings are atypical for hemangiomas.

**Figure 7:**
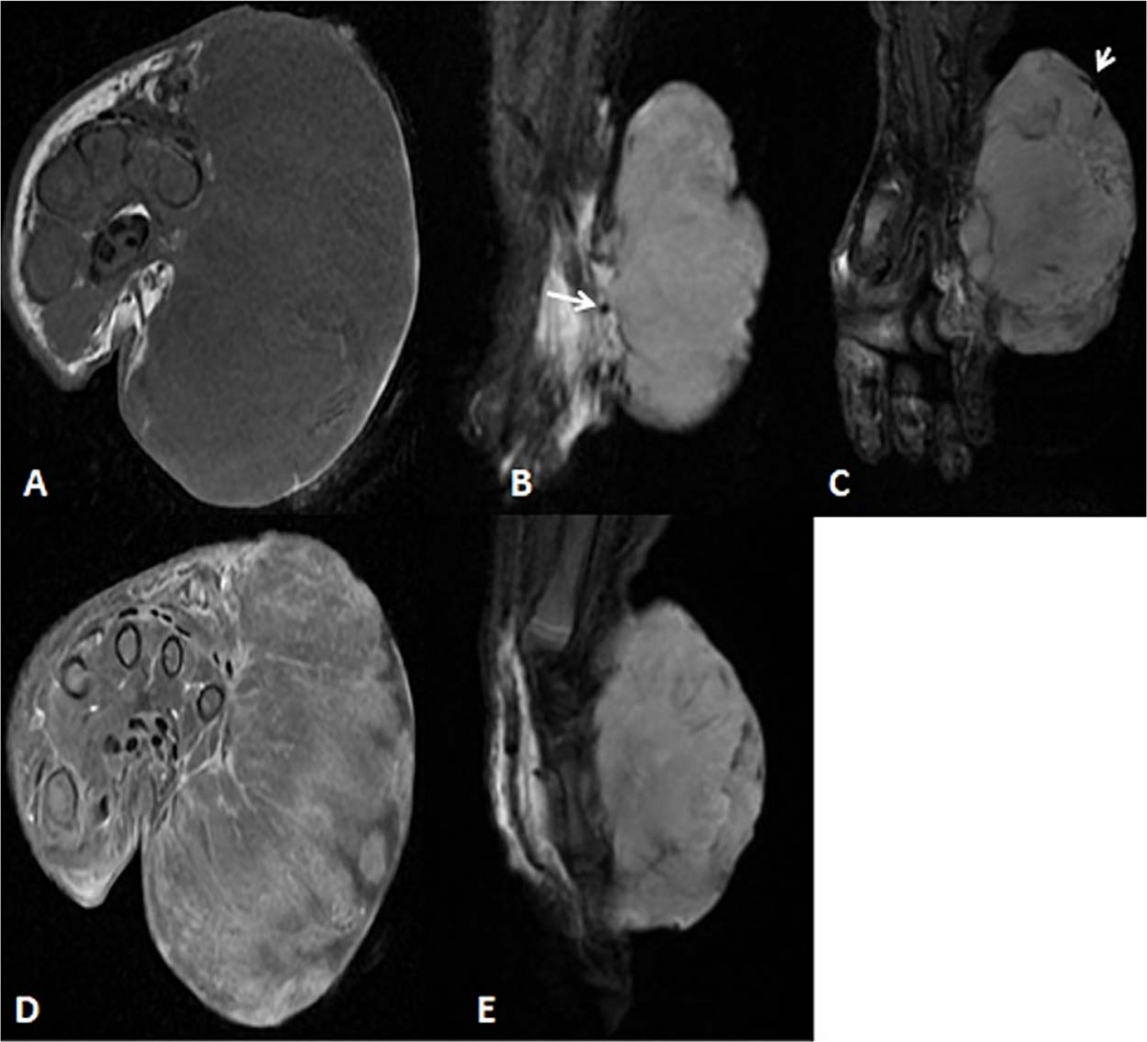
MRI of left hand mass. (A) Axial T1WI shows a huge homogenous isointense mass to the corresponding hand muscles signal intensity, which is seen along the medial aspect of left hand. (B and C) Coronal STIR demonstrate heterogeneous diffuse high signal intensity of the lesion and appears inseparable with sign of invasion to the fourth and fifth digits extensor compartment/tendons as well as presence of areas of signal void (arrow). (D and E) Axial and sagittal post-contrast *T*_1_ fat-saturated images show diffuse progressive intense enhancement of the lesion.

A 2-month-old girl was being followed for a suspected hemangioma on her left hand since birth. She was initially started on oral propranolol therapy but only received one dose, due to family concerns about possible side effects. The patient presented at the emergency room because of ulceration. It was observed clinically that the lesion had grown from 1.5 × 1.5 cm to 4 × 2 cm (Fig. 5). A biopsy of the lesion was taken. Ultrasound showed a partially defined large heterogeneously hyperechoic mass with internal vascularity on color Doppler with arterial and venous waveform giving atypical features of hemangioma (Fig. 6). Biopsy was done using FoundationOne Heme. The result showed stable microsatellite status and when we tested the tumor mutational burden, it showed two mutations per megabase. On MRI, a large lobulated subcutaneous soft tissue mass measuring 3.7 × 6.1 × 4.6 cm in dimensions was seen originating from the medial aspect of the left hand (Fig. 7). Internal flow voids, intermediate signal intensity on *T*_1_-weighted images, hyperintense signal on short tau inversion recovery (STIR)-weighted images, and noticeable heterogeneous enhancement on postcontrast sequences were all observed in the lesion. These imaging results raised suspicions of a malignant lesion.

**Figure 8:**
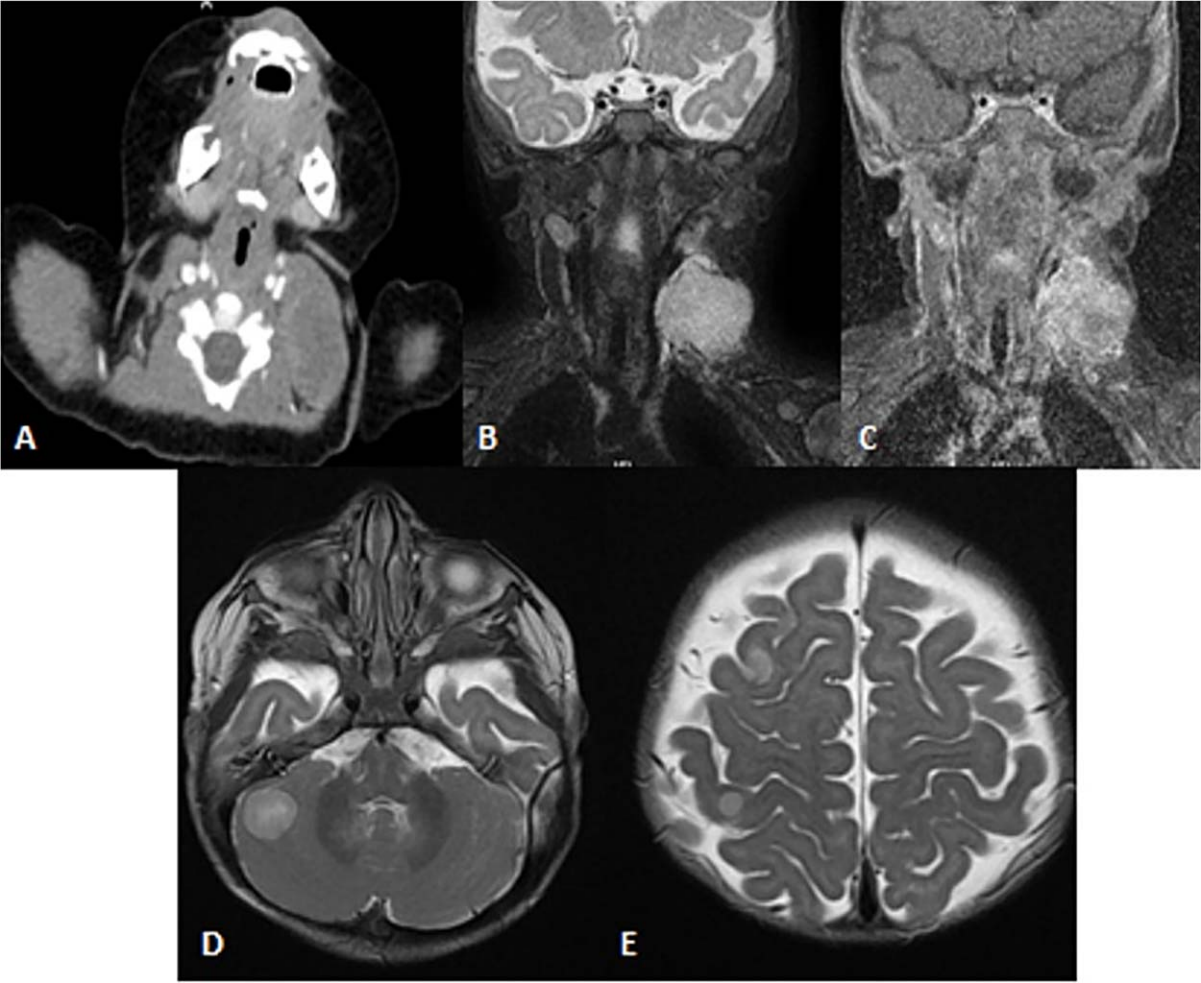
Patient underwent brain and neck MRI after incidental findings of left neck mass on chest CT. (A) Axial enhanced chest CT and the imaged part of the neck show a large heterogeneously enhanced left neck mass mostly arising from the left sternocleomastoid muscle with central necrosis causing mild mass effect upon left internal jugular vein with no sign of invasion or thrombosis. (B and C) Coronal STIR and postcontrast *T*_1_ fat-saturated images show homogenously high signal of the left neck mass arising from the left sternocleomastoid muscle on the STIR image with heterogeneous enhancement and central necrosis on the postcontrast image. (D and E) Axial T2WI images show multiple scattered, rounded high-signal-intensity supra- and infratentorial lesions at the gray–white matter junctions involving right frontoparietal lobes and the right cerebellar hemisphere, in keeping with brain metastasis.

Pathology showed a round blue cell; thus, chest, abdomen and pelvis CT was done, which showed two solid pulmonary nodules and a 3.8 × 2.1 × 3.4 cm and incidental findings of neck mass involving the left sternocleidomastoid muscle. Further assessment was done by neckMRI, which shows a homogenously high-signal-intensity mass arising from the left sternocleidomastoid muscle on STIR with heterogeneous enhancement and an area of central necrosis on postcontrast images. However, during neck MRI protocoling a right cerebellar mass was identified and further images of the brain were obtained. The brain MRIs showed multiple scattered rounded lesions that showed high signal intensity at the gray–white matter junction seen at right frontoparietal lobes and in the right cerebellar hemisphere, findings in keeping with brain metastasis (Fig. 8). To treat the hydrocephalus, an external ventricular drain was implanted. Unfortunately, the patient passed away as a result of brainstem metastases as the disease worsened.

## Discussion

Soft tissue sarcomas (STSs) are extremely rare tumors. The incidence is lower in pediatric patients, particularly in infants under 1 year of age, where it is estimated to be 1.6 per 100 000, and increases with age [7].

The clinical course of neonatal STSs can mimic and overlap those of benign vascular tumors like IHs, which hinders timely diagnosis and management of serious malignancies [4]. Infantile hemangiomas are the most common vascular tumors affecting children, with an incidence of 3%–10%. An IH is clinically diagnosed based on the onset, pattern of progression, and the presentation of a red lobulated plaque if superficial, or a blue/skin-colored subcutaneous mass occupying the deep dermis if it is a deep IH [8, 9]. Due to the high prevalence of IHs, the reliance on clinical assessment and history alone, and the absence of routine imaging and biopsy, IHs are easily confounded with STSs and other malignancies [4].

STSs are usually locally invasive but may metastasize to distant locations, most commonly the lungs, which is seen in our second case. These tumors are often present at birth and rapidly progress in size in the weeks following birth [7]. STSs affecting neonates are categorized into two groups: rhabdomyosarcomas (RMSs) and nonrhabdomyosarcoma soft tissue sarcomas (NRSTSs), with the former being more predominant than the latter but still rarely encountered. The most common STSs in neonates are RMSs, followed by fibrosarcomas, and malignant rhabdoid tumors [9]. NRSTSs are less common in neonates, but when found, are more aggressive [4].

Ultrasound and MRI are the first- and second-line imaging modalities, respectively, for evaluating lumps in children. However, imaging for STSs can be challenging, as their findings are often nonspecific and may mimic benign conditions [10]. Some malignancies may appear vascular on imaging, which can lead to misdiagnosis as hemangiomas. Therefore, the presence of vascularity alone should not be considered diagnostic of a hemangioma, especially when presenting with atypical signs and symptoms, and a biopsy should be pursued in the case of clinical suspicion [11].

This combined case report is distinctive from the existing literature because it documents two cases of rare malignancies, a round cell sarcoma that is positive for NF1 and an undifferentiated round blue cell sarcoma, masquerading as an IH even with imaging. They are not only rare, but they also scarcely affect infants. To our knowledge, there are only isolated case reports of such presentations and no combined report of two different sarcoma subtypes mimicking IHs in neonates has been made to date.

In Saudi Arabia, only a single case has been published involving a RMS of the nasal cavity initially diagnosed as a hemangioma. The lesion was first noticed at 4 months of age and managed for ~2 years with several therapies aimed at IH, such as oral and intravenous steroids, and percutaneous embolization. These treatments led to only temporary improvement and a rapid relapse. A diagnosis of RMS was not established until the patients reached 2 years of age [12]. This case demonstrates the challenges faced when diagnosing and treating STSs, emphasizing the need for early detection.

There are only a few cases of sarcomas mimicking IHs in neonates documented in international published work. One such case involved a 6-month-old female who was initially thought to have an IH and was subsequently treated as such. The lesion drastically enlarged and ulcerated, which raised suspicion about the lesion being a case of an atypical hemangioma, with imaging backing the said diagnosis [13]. The true diagnosis of an infantile fibrosarcoma was not made until a biopsy was taken [13]. Hu *et al.* and Bellfield and Beets-Shay documented similar cases of an infantile fibrosarcoma in a 4-month-old and a 1-week-old girl, respectively [14, 15].

Such cases highlight the importance of increased vigilance when dealing with presumed infantile and congenital hemangiomas, especially those with an unusual clinical course. Early application of advanced imaging modalities and timely biopsies must be considered in the event that clinical manifestations stray from the typical IH course. There should be a multidisciplinary approach to avoid delays in diagnosis and ensure better outcomes for the patients [4, 10, 11, 14, 15].
