# Correction to: Is it a hemangioma? A combined case report of soft tissue sarcomas mimicking infantile hemangioma

**DOI:** 10.1093/jscr/rjaf844

**Published:** 2025-10-11

This is a correction to: Shaden Almutairi (*et al*), Is it a hemangioma? A combined case report of soft tissue sarcomas mimicking infantile hemangioma, *Journal of Surgical Case Reports*, Volume 2025, Issue 9, September 2025, https://doi.org/10.1093/jscr/rjaf748

The following changes have been made to the originally published manuscript. The last name of the second author has been emended to read Wed Al Wabel.

